# Myocardial Infarction With Non-obstructive Coronary Arteries (MINOCA): A Case Report and Comprehensive Discussion of Pathophysiology and Risk Factors

**DOI:** 10.7759/cureus.67144

**Published:** 2024-08-18

**Authors:** Pawel Borkowski, Maisha Maliha, Michal Borkowski, Natalia Borkowska, Nikita Singh, Abhyuday Chauhan, Ishmum Chowdhury, Shreyas Yakkali, Vikyath Satish, Hansol Choi

**Affiliations:** 1 Internal Medicine, Albert Einstein College of Medicine, Jacobi Medical Center, Bronx, USA; 2 Medicine, Private Practice, Wroclaw, POL; 3 Pediatrics, Samodzielny Publiczny Zakład Opieki Zdrowotnej (SPZOZ), Krotoszyn, POL

**Keywords:** pathophysiology, non-obstructive coronary arteries, risk factors, cardiac troponin, myocardial infarction with non-obstructive coronary arteries (minoca)

## Abstract

Myocardial infarction with non-obstructive coronary arteries (MINOCA) refers to the occurrence of myocardial infarction symptoms and signs despite angiographic findings showing normal or near-normal coronary arteries. Unlike the more commonly recognized myocardial infarction with coronary artery disease (MICAD), MINOCA often has a better prognosis; however, it is not without risk, as it is associated with increased mortality. We present a 72-year-old female who presented to the hospital with acute chest pain. Following a thorough diagnostic workup, including laboratory tests, left heart catheterization, and cardiac imaging, she was diagnosed with MINOCA. This case report provides a comprehensive review of the pathophysiological mechanisms underlying MINOCA, such as plaque disruption without significant stenosis, microvascular dysfunction, coronary artery spasm, coronary thrombosis or embolism, and spontaneous coronary artery dissection. Additionally, we explore the associated risk factors, highlighting the unconventional risk factors. MINOCA represents a diverse clinical condition with various causes and complex pathophysiology. The variability underscores the necessity for further research to deepen our understanding of this condition. Enhanced knowledge will lead to better diagnostic and treatment strategies, ultimately improving patient outcomes.

## Introduction

Heart disease is the leading cause of death for men, women, and most ethnic groups, with 702,880 deaths in 2022 [1]. In 2019, heart disease prevalence increased with age: 1.0% in adults aged 18-44, 3.6% in those 45-54, 9.0% in those 55-64, 14.3% in ages 65-74, and 24.2% in those 75 and older [2]. Acute coronary syndrome (ACS) includes ST-segment elevation myocardial infarction (STEMI), non-ST-segment elevation myocardial infarction (NSTEMI), and unstable angina (UA). ACS affects over 780,000 people in the United States annually, with approximately 70% of cases being NSTEMI/UA [3]. Key risk factors include smoking, hypertension, diabetes, hyperlipidemia, male sex, physical inactivity, and obesity [4,5]. ACS typically results from coronary artery disease (CAD), which is characterized by the accumulation of cholesterol plaques in the coronary arteries. These plaques can erode or rupture, leading to the formation of a thrombus and causing significant obstruction to blood flow to the heart [6]. However, ACS can also occur without substantial plaque buildup. In such cases, it is referred to as myocardial infarction with non-obstructive coronary arteries (MINOCA), which accounts for 5-15% of all myocardial infarction (MI) cases [7-11].

MINOCA is defined by the presence of clinical symptoms and signs of MI despite angiographic findings of normal or near-normal coronary arteries [12]. The criteria include having an active MI, having non-obstructive coronary arteries (no stenosis ≥50%), and lacking a clinically evident specific cause for the acute presentation [13]. Therefore, the diagnosis is primarily established through a clinical history, physical examination, electrocardiography, cardiac enzyme measurement, echocardiography, left heart catheterization (LHC), provocative testing for coronary spasm, and cardiovascular magnetic resonance imaging (CMR). In the past, MINOCA was considered benign compared to MI with obstructive coronary artery disease (MICAD), fewer cardiovascular risk factors among patients with MINOCA, and the presence of non-obstructive coronary arteries [14]. In fact, MINOCA patients have lower all-cause mortality than MICAD, with 63% lower in-hospital and 41% lower 12-month mortality [9]. Nevertheless, a 12-month all-cause mortality rate of 4.7% (95% CI, 2.6%-6.9%) for MINOCA patients indicates a guarded prognosis, though outcomes can vary based on the underlying causes of MINOCA [9].

It is important to note that while some authors consider myocarditis and Takotsubo syndrome (TS) as part of the MINOCA spectrum, there is increasing consensus to exclude these conditions [15]. Although these conditions share symptoms with MI - such as chest pain, elevated serum troponin levels, and ECG changes - their underlying pathologies lack an ischemic component [15,16]. CMR imaging is valuable in distinguishing between MI, myocarditis, and TS [17]. Moreover, it is essential to rule out other potential cardiac causes of chest pain or elevated serum troponin levels, such as pulmonary embolism or tachyarrhythmias [15,18-20].

## Case presentation

**Figure 1:**
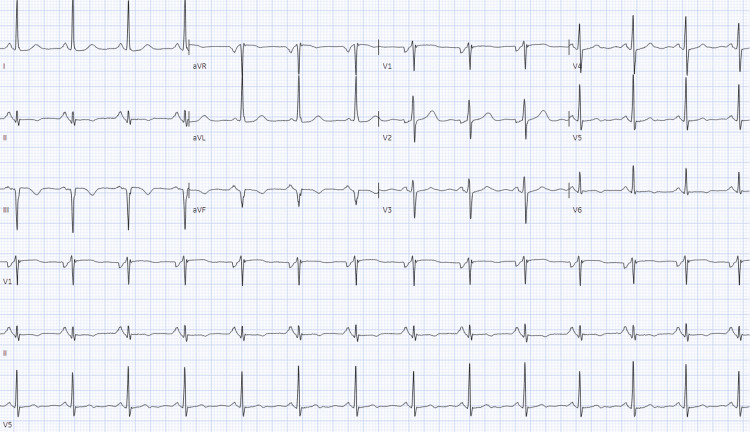
EKG - Normal sinus rhythm with a ventricular rate of 82 beats per minute, a normal axis, and T wave inversions in the inferolateral leads.

A 72-year-old female with a medical history of hypertension, hyperlipidemia, and schizophrenia presented to the emergency department due to chest pain. Her medications included lisinopril 40 mg, nifedipine 90 mg, simvastatin 40 mg, and risperidone 1 mg. The patient reported that her chest pain, which began several hours prior to presentation, was substernal, excruciating, and radiated to her back. The pain was accompanied by nausea and vomiting. She noted that she had experienced intermittent chest pain over the past few months, often exacerbated by emotional stress. She did not monitor her blood pressure at home and reported constant stress in her domestic environment. The patient denied palpitations, shortness of breath, or a family history of heart disease. On examination, her vital signs were: temperature 36.2°C, blood pressure 178/104 mmHg, pulse rate 67 beats/minute, and respiratory rate 17 breaths/minute. Pulse oximetry showed a blood oxygen saturation of 98%. Physical examination revealed normal heart sounds without rubs, murmurs, or gallops, clear lung fields, absent hepatojugular reflex, and no peripheral edema. Laboratory results (Table 1) showed hemoglobin at 10.8 mg/dL, normal kidney and liver function tests, C-reactive protein (CRP) at 2.3 mg/L, N-terminal pro-brain natriuretic peptide at 335 pg/mL, and high-sensitivity cardiac troponin T at 49 ng/L. The EKG demonstrated normal sinus rhythm with a ventricular rate of 82 per minute, a normal axis, and T wave inversions in the inferolateral leads (Figure 1). A chest CT scan ruled out aortic dissection and other acute chest pathologies.

**Table 1:** Laboratory results.

Laboratory Test	Actual Result	Normal Range
White blood cells (WBC)	7.47 K/uL	3.8 – 10.5 K/uL
Neutrophils (absolute)	6.53 K/uL	1.8 – 7.4 K/uL
Lymphocytes (absolute)	0.7 K/uL	1 – 3.3 K/uL
Hemoglobin (HGB)	10.8 g/dL	11.5 – 15.5 g/dL
Platelets (PLT)	267 K/uL	150 – 400 K/uL
Creatinine (Cr)	0.9 mg/dL	0.5 – 1.3 mg/dL
Blood urea nitrogen (BUN)	15 mg/dL	22 – 31 mg/dL
Alanine aminotransferase (ALT)	11 U/L	10 – 45 U/L
Aspartate transferase (AST)	23 U/L	10 – 40 U/L
Lipase	34 U/L	7 – 60 U/L
Troponin T (High sensitivity)	49 ug/L	0 – 0.14 ng/L
Creatine kinase (CK)	252 U/L	5 – 150 U/L
Pro B-type natriuretic peptide	335 pg/mL	1 – 125 pg/mL
C-reactive protein (CRP)	2.3 mg/L	0 – 5 mg/L
HIV-1/HIV-2 antigen/antibody	Nonreactive	Nonreactive
Prothrombin time (PT)	11.8 seconds	9.4 – 12.5 seconds
Activated partial thromboplastin clotting time (aPTT)	27.6 seconds	25.1 – 36.5 seconds

**Figure 2:**
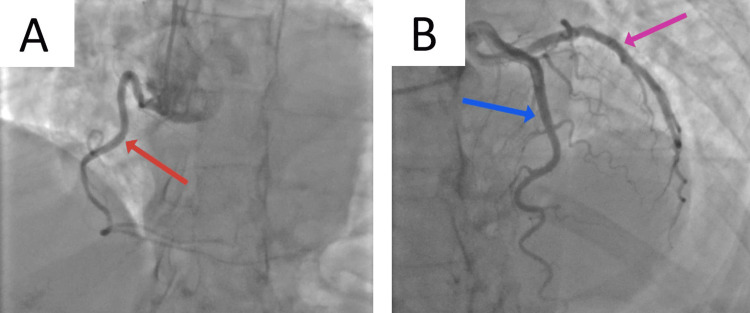
Coronary angiogram. Red Arrow - The right coronary artery (RCA) Blue Arrow - The left circumflex coronary artery (LCX) Purple Arrow - The left anterior descending artery (LAD)

**Figure 3:**
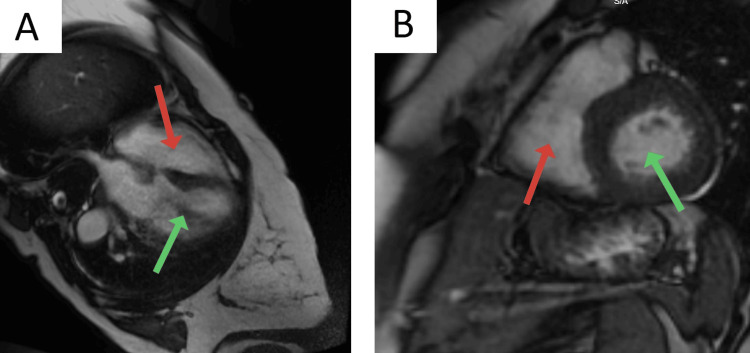
Cardiac Magnetic Resonance Imaging. A: Long axis. Red arrow: Right ventricle; Green arrow: Left ventricle B: Short axis. Red arrow: Right ventricle; Green arrow: Left ventricle

The patient was started on an NSTEMI protocol, including loading doses of aspirin and clopidogrel, and a heparin drip was started. The following day, an official echocardiogram showed normal biventricular function with a left ventricular ejection fraction of 75%, mild left ventricular hypertrophy, no valvular pathology, and no wall motion abnormalities (Videos 1, 2). During the hospital course, the patient's serum troponin levels increased from an initial 49 ng/L (midnight) to 92 ng/L (3 am), 118 ng/L (6 am), 1436 ng/L (3 pm), and eventually 1597 ng/L (7 pm); however, the EKG did not reveal new ischemic changes. A left heart catheterization (LHC) was performed, which revealed normal coronary arteries, raising suspicion for MINOCA (Figure 2). The patient was subsequently monitored in the telemetry unit. With the trending down of serum troponin levels, she was started on verapamil and isosorbide dinitrate, showing an excellent response (no further chest pain episodes). A CMR was performed and did not reveal any significant pathology (Figure 3). The patient was discharged in stable condition with a plan for close cardiology follow-up.

**Video 1:** Echocardiogram - Parasternal Long Axis.

**Video 2:** Echocardiogram - Parasternal Short Axis.

## Discussion

The patient presented with chest pain and a gradual rise in troponin levels, but notably without new ischemic changes on her EKG and with normal coronary arteries on catheterization. These findings guided us toward a diagnosis of MINOCA. A key factor in this patient's presentation was her history of schizophrenia and chronic domestic stress, which could contribute to vasospastic angina. Her clinical response to verapamil and isosorbide dinitrate strongly supports the role of coronary vasospasm in her presentation. This therapeutic approach, aimed at alleviating vasospasm, effectively prevented further episodes of chest pain. This case emphasizes the importance of evaluating patients with non-obstructive coronary arteries for MINOCA. It highlights the need to explore mechanisms of myocardial injury beyond the traditional plaque rupture or erosion with subsequent thrombosis seen in obstructive CAD. In the following discussion, we will explore the underlying pathophysiological mechanisms and the risk factors contributing to MINOCA.

Main pathophysiologic mechanisms

MINOCA is a unique syndrome with a wide spectrum of underlying pathophysiologic mechanisms [21]. The etiology of MINOCA can be classified into atherosclerotic (plaque disruption) and non-atherosclerotic (microcirculatory dysfunction, vasospasm, coronary thrombosis or embolism, and spontaneous coronary artery dissection [SCAD]) causes of myocardial necrosis, or alternatively, into microvascular and epicardial causes [7,22].

Plaque disruption includes plaque rupture (fibrous cap break, connection between the plaque cavity and the artery lumen), plaque erosion (thrombus on the plaque surface without rupture), and calcific nodules [7]. These lesions often show a large lipid core and thin fibrous cap [22]. Plaque disruption can lead to thrombus formation and MI via distal embolization, local thrombosis with subsequent thrombolysis, or artery spasm [7,23]. Intravascular ultrasound (IVUS) detects plaque disruption in around 40% of patients with MINOCA [24].

The large coronary arteries usually contribute less than 10% to coronary vascular resistance, becoming significant only with over 70% obstruction [25]. In comparison, the coronary microvasculature (<0.5 mm diameter) accounts for over 70% of resistance under normal conditions [25]. Coronary microvascular dysfunction is detected in 30% to 50% of patients with chest discomfort and nonobstructive CAD [7]. Microvascular angina is defined by ischemic chest discomfort, nonobstructive coronary arteries, and impaired coronary flow (1) coronary flow reserve under 2.0 with adenosine; 2) microvascular spasm indicated by chest pain and ischemic ECG changes during acetylcholine testing without epicardial spasm; or (3) poor coronary blood flow proven by the delayed passage of the contrast) [7]. The underlying pathophysiology of microvascular dysfunction is unclear, but the proposed mechanisms include high vascular tone, coronary microembolization, abnormal nitric oxide production, and inflammatory response [26].

Vasospastic angina (variant angina, Prinzmetal angina) involves symptoms at rest and ST elevation on ECG, usually in the early morning during reduced vagal tone, and is often associated with over 90% stenosis in a proximal coronary segment seen on LHC [27]. Diagnosing vasospastic angina requires documenting coronary artery spasm with intracoronary acetylcholine administration [27]. The mechanisms for coronary artery spasm include endothelial dysfunction, hyperreactivity of vascular smooth muscle cells, local fibromuscular hyperplasia, hyperadrenergic state, excessive release of vasoconstrictor substances, and dysregulated calcium homeostasis [28,29].

MINOCA can result from coronary thrombosis or embolism affecting the microcirculation or from partial lysis of an epicardial thrombus, leading to nonobstructive angiographic findings [7]. Among inherited hypercoagulable disorders, Factor V Leiden is the most extensively studied and most strongly associated with MINOCA [9]. The prevalence of Factor V Leiden in the USA varies among different populations: 5.2% among White Americans, 2.2% among Hispanic Americans, 1.2% among African Americans, 0.45% among Asian Americans, and 1.25% among Native Americans [30]. Pasupathy et al. conducted a comprehensive systematic review and found that 12% of MINOCA patients had Factor V Leiden, a prevalence significantly higher than that observed in the general population [9]. Other hereditary causes include antithrombin deficiency, protein C deficiency, protein S deficiency, and hyperhomocysteinemia [31]. A hypercoagulable state can cause MINOCA by inducing inflammatory responses that lead to coronary artery spasm, endothelial dysfunction, and platelet aggregation [32,33]. It can cause the formation of microthrombi in the coronary microvasculature and may also lead to embolic events where clots from other parts of the body migrate to the coronary arteries, such as in cases of non-bacterial thrombotic endocarditis or infective endocarditis [34-37].

SCAD involves the separation of coronary artery walls, leading to the formation of an intramural hematoma [38,39]. This can expand and obstruct coronary blood flow. SCAD is an emerging cause of ACS in young females [40]. The etiopathogenesis of this condition is unknown, but it may be linked to fibromuscular dysplasia, excess of estrogen or progesterone, physical stress, and mental health problems (e.g. depression, anxiety, and post-traumatic stress disorder) [38,41].

Conventional and unconventional risk factors

MINOCA patients differ from those with MICAD in demographic and clinical characteristics. The average age for MINOCA patients is 58 years, compared to 61 years for MICAD (p < 0.001), though some studies report an average of 61 years for MINOCA [9,11,42]. Women are disproportionately affected by MINOCA, with prevalence rates between 43-77%, as opposed to 24-41.5% in MICAD (p < 0.001) [9,43]. The disparities in the prevalence and outcomes of cardiovascular disease (CVD) in general are well-documented, with Black and Hispanic populations often being disproportionately disadvantaged [44,45]. Racial disparities are also evident between MINOCA and MICAD populations, with higher rates of MINOCA observed among Black individuals (10.2% vs. 6.4%; P < 0.001) and Hispanic individuals (10.4% vs. 7.1%; P = 0.022) compared to MICAD [43,46]. Interestingly, conventional cardiovascular risk factors are less prevalent among patients with MINOCA compared to those with MICAD. For instance, the prevalence rates of hypertension (54.9% vs. 67.2%, P = 0.001), diabetes mellitus (17.4% vs. 31.6%, P = 0.001), dyslipidemia (54.9% vs. 69.6%, P = 0.001), recent smoking (34.5% vs. 60.3%, P = 0.001), and obesity (42.1% vs. 54.1%, P = 0.001) are significantly lower in MINOCA patients [46].

There is growing evidence linking cancer to MINOCA. For instance, an extensive systematic review and meta-analysis by Pelliccia et al. reported that 655 of 26,636 patients with MINOCA (2.5%) had a cancer diagnosis at presentation [47]. Additionally, Stepien et al. found that active cancer was more frequently observed in patients with MINOCA (29.2%) compared to those with MICAD (12.0%), with the difference being statistically significant (p < 0.001) [48]. Neoplasms and chemotherapy can induce coronary artery spasm, hypercoagulable state, endothelial damage, and acute thrombosis by releasing inflammatory and angiogenic cytokines and interacting with adhesion molecules [49,50]. Additionally, the risk of MI increases when chemotherapy agents from different classes are used together [49]. Special consideration is necessary for nephrotoxic chemotherapeutic agents like cisplatin [51]. In addition to the previously mentioned mechanisms, these medications pose additional risks. A study by Zalewska-Adamiec et al. demonstrated that MINOCA patients with an eGFR < 60 mL/min/1.73 m² had a significantly higher three-year mortality rate compared to those with an estimated glomerular filtration rate (eGFR) ≥ 60 mL/min/1.73 m² (33.96% vs. 9.6%, p < 0.0001) [52].

Some studies suggest that pro-inflammatory disorders may be risk factors for developing MINOCA. For example, Espinosa et al. reported that patients with MINOCA had higher rates of connective tissue disorders (5.8% vs. 1.4%, p = 0.01) and autoimmune diseases (14.5% vs. 7.8%, p = 0.058) than patients with MICAD [53]. In addition, there is a recognized link between antiphospholipid syndrome and MINOCA [54,55]. Increased oxidative stress and low-grade systemic inflammation are associated with myocardial dysfunction and have been proposed as risk factors, potentially leading to microvascular dysfunction [56,57]. Similar mechanisms are likely involved in developing MINOCA in patients with systemic infections. For example, severe acute respiratory syndrome coronavirus 2 (SARS‑CoV‑2) has been reported as a cause of MINOCA [58]. The SARS-CoV-2 virus is known to cause small vessel vasculitis and microvascular thrombosis, suggesting a possible pathophysiologic mechanism [59]. However, further research is needed to explore whether this mechanism applies to the MINOCA population.

The relationship between CVD and mental health is bidirectional, where CVD can lead to mental health disorders, and mental health disorders can worsen or increase the likelihood of CVD [60]. The link between mental health disorders and MINOCA is less well understood. However, growing evidence suggests that mental health disorders may be associated with MINOCA and could act as risk factors [61]. For example, Gu et al. identified new-onset depression as a prognostic factor for all-cause mortality (HR: 7.250, 95% CI: 4.735-11.100, P < 0.001) and cardiovascular events (HR: 3.411, 95% CI: 2.490-4.674, P < 0.001) in patients with MINOCA [62]. Additionally, there is some evidence that anxiety disorders may be linked to worse outcomes in MINOCA patients. He et al. found that anxiety was a prognostic factor for all-cause mortality (HR: 1.547, 95% CI: 1.006-2.380, P = 0.04) and major adverse cardiovascular events (HR: 1.460, 95% CI: 1.049-2.031, P = 0.025) among MINOCA patients [63]. Psychiatric disorders may contribute to MINOCA through stress-related mechanisms, such as heightened sympathetic activity and increased levels of stress hormones, which can lead to vasoconstriction and inflammation [64]. Moreover, unhealthy behaviors often associated with these disorders, such as poor diet and smoking, may further elevate cardiovascular risk.

Pregnancy can reveal underlying CVD in women, with pregnancy-associated myocardial infarction (PAMI) occurring in 2.8 to 8.1 per 100,000 deliveries [65-67]. Although MI during pregnancy is rare, it poses significant risks of maternal, fetal, and neonatal complications [68]. Key risk factors include maternal age over 35, smoking, hypertension, hyperlipidemia, and diabetes mellitus [68-70]. The etiologies of PAMI are diverse. A SCAD is a leading cause, accounting for up to 43% of cases [71]. Other causes include MICAD at 27% and coronary thrombus without evident atherosclerosis at 17% [71]. Hormonal factors, such as elevated levels of progesterone and estrogen, contribute to SCAD by compromising the integrity of arterial walls and enhancing matrix metalloproteinase activity [72]. Additionally, pregnant women may experience coronary spasm due to endothelial dysfunction and increased vascular reactivity from ergot derivatives administration [72,73]. Lastly, pregnancy is linked to reduced tissue plasminogen activator activity and elevated levels of coagulation factors, increasing the risk of thrombosis [73].

Research indicates that heavy alcohol consumption is associated with an increased risk of ischemic heart disease and hypertension, while these effects are not observed with low to moderate drinking [74,75]. However, there is limited data on alcohol as a risk factor for MINOCA or its impact on MINOCA patients. However, there is evidence that alcohol drinking might negatively affect coronary microvasculature through mechanisms such as suppression of angiogenesis or causing increased thickness of coronary microvessels [76,77]. In addition, Lee et al. observed that among the Korean population with MINOCA, male patients exhibited significantly higher alcohol consumption than females, suggesting a potential link between ethanol use and MINOCA in men [78]. These observations suggest that alcohol might not be neutral for MINOCA patients, but further studies are needed to examine this relationship.

## Conclusions

While MINOCA generally presents with a better prognosis compared to MICAD, it remains a condition with significant clinical implications and mortality risk. Various pathophysiologic mechanisms, including plaque disruption, coronary microvascular dysfunction, vasospasm, thrombosis, and spontaneous coronary artery dissection, contribute to the MINOCA spectrum. Additionally, conventional cardiovascular risk factors like hypertension, diabetes, and dyslipidemia are less prevalent in MINOCA compared to MICAD, suggesting a unique patient profile. Emerging evidence also points to unconventional risk factors, such as cancer, inflammatory conditions, mental health disorders, pregnancy, and heavy alcohol consumption, as significant contributors. Future research should focus on the mechanisms underlying these risk factors and developing targeted therapeutic interventions. By enhancing our understanding of MINOCA, we can improve diagnostic accuracy, optimize treatment protocols, and enhance patient outcomes.
